# Test–retest reliability and measurement error of the WHO-5 Well-being Index and the Problem Areas in Diabetes questionnaire (PAID) used in telehealth among patients with type 1 diabetes

**DOI:** 10.1186/s41687-022-00505-3

**Published:** 2022-09-23

**Authors:** Liv Marit Valen Schougaard, Tinne Laurberg, Kirsten Lomborg, Troels Krarup Hansen, Niels Henrik Hjollund, Annesofie Lunde Jensen

**Affiliations:** 1 AmbuFlex - Center for Patient-Reported Outcomes, Central Denmark Region, Gødstrup Hospital, Møllegade 16, 7400 Herning, Denmark; 2 Steno Diabetes Centre Aarhus, Aarhus University Hospital, Hedeager 3, 8200 Aarhus N, Denmark; 3 Steno Diabetes Centre Copenhagen, Borgmester Ib Juuls Vej 83, 2730 Herlev, Denmark; 4 Department of Clinical Medicine, University of Copenhagen, Blegdamsvej 3B, 2200 Copenhagen N, Denmark; 5 Department of Clinical Medicine, Aarhus University, Palle Juul-Jensens Boulevard 82, 8200 Aarhus N, Denmark; 6 Department of Clinical Epidemiology, Aarhus University Hospital, Olof Palmes Allé 43-45, 8200 Aarhus N, Denmark; 7 ResCenPI – Research Centre for Patient Involvement, Aarhus University and the Central Denmark Region, Palle Juul-Jensens Boulevard 99, 8200 Aarhus N, Denmark

**Keywords:** Patient-reported outcome measures, Psychometrics, Reproducibility of results, WHO-5 Well-being Index, Problem Areas in Diabetes, Diabetes mellitus Type 1

## Abstract

**Background:**

Patient-reported outcome (PRO) measures may be used in telehealth for the clinical assessment of mental health and diabetes distress, which are important aspects in diabetes care, but valid and reliable instruments on these topics are necessary. We aimed to evaluate the test–retest reliability and measurement error of the Danish versions of the WHO-Five Well-being Index (WHO-5) and Problem Areas in Diabetes (PAID) questionnaires used in a PRO-based telehealth intervention among patients with type 1 diabetes. A further aim was to evaluate the test–retest reliability of single items concerning patients’ symptom burden and general health status.

**Methods:**

Outpatients with type 1 diabetes from the Steno Diabetes Center Aarhus, Aarhus University Hospital, Denmark, were enrolled from April 2019 to June 2020. Patients aged ≥ 18 who had type 1 diabetes for > 1 year, internet access, and the ability to understand, read, and write Danish were included. Intraclass correlation coefficients (ICC) and weighted Kappa values were used to assess test–retest reliability, and measurement error was assessed by estimating the minimal detectable change (MDC).

**Results:**

A total of 146/255 (57%) patients completed the web questionnaire twice. The median response time between the two-time points was five days. The ICC of the WHO-5 scale was 0.87 (95% CI 0.82–0.90), and MDC was 18.56 points (95% CI 16.65–20.99). The ICC of the PAID scale was 0.89 (95% CI 0.84–0.92), and MDC was 11.86 points (95% CI 10.46–13.70). Overall, test–retest reliability of single symptoms and general health status items was substantial.

**Conclusions:**

The WHO-5 and PAID questionnaires, and single symptoms and general health status items showed substantial test–retest reliability among patients with type 1 diabetes. Measurement error of the PAID questionnaire was considered acceptable; however, a larger measurement error of the WHO-5 questionnaire was observed. Further research is recommended to explore these findings.

## Background

Remote monitoring using patient-reported outcome (PRO) measures is becoming more convenient and useful as telehealth technologies develop, allowing new opportunities, such as real-time monitoring of symptoms and flexible scheduling of hospital appointments [1, 2]. Thus, PRO measures in telehealth could contribute to the reorganization of the healthcare system for follow-up activities in patients with chronic conditions by prioritizing or optimizing the use of healthcare resources and promoting patient-centered care [3, 4].

In patients with diabetes, face-to-face consultations are traditionally used in clinical care; however, telehealth initiatives that provide more flexible and convenient services are increasingly adopted [5]. In Denmark, a PRO-based telehealth initiative called *DiabetesFlex* has been developed for patients with type 1 diabetes [6]. In DiabetesFlex, patients fill in a web-based questionnaire at home, and healthcare professionals use the patient’s PRO data to identify whether patients need further clinical attention. The impact of the DiabetesFlex intervention was evaluated in a randomized design, demonstrating no differences in clinical outcomes, better well-being, and lower diabetes distress than standard face-to-face visits [7]. As of August 2022, PRO-based telehealth/DiabetesFlex has been offered and accepted by 335 outpatients with type 1 diabetes from one outpatient clinic in the Central Denmark Region and will soon be adopted in other clinics in the region and expanded to patients with type 2 diabetes.

The selection of PRO measures is central to a PRO-based telehealth solution, such as DiabetesFlex, and a disease-specific questionnaire has been developed in close cooperation with patients and clinical experts to ensure content and face validity [6]. A PRO measure must be used according to its purpose, measurement properties such as validity and reliability must be considered, and users must know how to interpret the PRO measure’s results [8]. Among the significant aspects of diabetes care is the assessment of mental health and diabetes distress; hence, the WHO-Five Well-being Index (WHO-5) and Problems Areas in Diabetes (PAID) questionnaires were selected in the PRO-based telehealth solution DiabetesFlex.

The WHO-5 is a five-item generic questionnaire measuring mental well-being during the last 2 weeks. WHO-5 was originally developed for patients with diabetes but has been applied across several patient populations and countries [9, 10]. The psychometric properties of the WHO-5 scale have been described in terms of construct validity, predictive validity, and internal consistency in several patient populations, including diabetes and a Danish context [9]. Factor analyses have confirmed a one-factor structure of the WHO-5 scale [9]. However, studies investigating the test–retest reliability and/or measurement error of the WHO-5 have only been explored in a few other patient populations, e.g. in epilepsy and rheumatoid arthritis [11–13]. Only one study has reported the WHO-5's measurement error in an epilepsy population [13]. Thus, further research on this topic is necessary for other patient populations, including diabetic patients.

Moreover, we selected PAID which is a 20-item disease-specific questionnaire measuring diabetes distress, for example, feeling scared about living with diabetes, feelings of deprivation regarding food and meals, and worrying about low blood sugar reactions [14]. PAID has been widely used, and its measurement properties for construct and convergent validity, internal consistency, and responsiveness have been evaluated, including in a Danish context [15–22]. PAID was originally conceptualized as a large general factor, and summation of the 20 PAID items into a total score was recommended [14]. Subsequent studies have found both a two- and four factor structure [23, 24], but other studies have shown mixed results [19]. From a clinical perspective, the total score of 20 items have been found to have sufficient clinical sensitivity in detecting diabetes-related distress [25]. Few studies, however, have assessed test–retest reliability [19, 26], and no studies reporting measurement error of the PAID scale has been identified. The lack of research regarding test–retest reliability and measurement error of the PAID scale was recently pointed out as an issue in a systematic review [22]. Thus, we consider the need to investigate the PAID scale’s reliability and measurement error.

This study’s aim was to evaluate the test–retest reliability and measurement error of the Danish WHO-Five Well-being Index (WHO-5) and the Danish Problem Areas in Diabetes (PAID) questionnaire used in a PRO-based telehealth solution (DiabetesFlex) among patients with type 1 diabetes. A further aim was to evaluate the test–retest reliability of single items concerning patients’ symptoms and general health status.

## Methods

### Study participants and setting

We conducted a test–retest reliability study among outpatients with type 1 diabetes from the Steno Diabetes Center Aarhus, Aarhus University Hospital, Denmark. Patients aged at least 18 who had type 1 diabetes for > 1 year, internet access, and the ability to understand, read, and write Danish were included from April 2019 to June 2020. The included patients were enrolled in the PRO-based telehealth intervention DiabetesFlex [6]. The patients filled in a questionnaire at two-time points. First, they completed the annual DiabetesFlex questionnaire before a scheduled appointment at the department (Test 1). Two reminders were sent to non-responders. Second, the patients completed the same questionnaire approximately 5 days later (Test 2). No reminders were sent to non-responders of Test 2. At both time points, the questionnaires and study information were sent to the patients electronically via “e-box,”—a secure electronic mailbox available for all Danish citizens. Moreover, the patients completed the questionnaires electronically at both time points.

### The DiabetesFlex questionnaire

The DiabetesFlex questionnaire includes information specific to aspects of daily life with diabetes, using several generic scales and items, for example, the WHO-Five Well-being Index (WHO-5) [9, 10], the Problem Areas in Diabetes (PAID) scale [14], and items from the Short Form 36 Health Survey (SF-36) [27]. WHO-5 has a unidimensional structure and comprises five positively worded items with six ordinal response categories ranging from 0 “At no time” to 5 “All of the time.” The total percentage score ranges from 0 to 100, and a score of ≤ 50 indicates impaired well-being and depression risk [9]. PAID comprises 20 negatively worded items with five ordinal response categories ranging from 0 “Not a problem” to 4 “Serious problem.” The total percentage score ranges from 0 to 100, and a score above 40 indicates emotional burnout and a risk of diabetes distress [14]. Two items from SF-36 were included: “In general, would you say your health is: excellent, very good, good, fair, or poor” and “Compared to 1 year ago, how would you rate your health in general now?” with the response categories: “Much better now than 1 year ago/Somewhat better now than 1 year ago/About the same/Somewhat worse now than 1 year ago/Much worse than 1 year ago” [27].

In addition, some ad hoc items to determine patients’ symptom status have been developed in close cooperation with patients and clinical experts, for example, dyspnea, rapid heart rate, chest pain, foot ulcer, and feet pain. These items have five ordinal response categories, ranging from “Never” to “Very often.” Also, the DiabetesFlex questionnaire includes questions about blood pressure, weight, diabetes eye and foot care, and a list of diabetes-related topics, for example, measurement of blood sugar, nutritional issues, and daily life with diabetes, that the patients can tick off if they want to talk about the topic during the next consultation at the hospital. Finally, patients can leave a comment if they have anything else to add that is unaddressed by the questionnaire’s selected items. The mean time to complete the DiabetesFlex questionnaire electronically was estimated to be 13 min.

### Statistical analysis

A sample size of at least 50 participants was considered sufficient according to the Consensus-based Standards for the Selection of health Measurement Instruments (COSMIN) checklist for studies assessing validity and reliability [28, 29]. The interval between Test 1 and Test 2 was estimated by calculating the days between the two-response time point dates. Descriptive data were presented for patient characteristics and for each item in the WHO-5 and PAID questionnaires to determine the extent of floor and ceiling effects. Internal consistency of the WHO-5 and PAID scales was evaluated by estimating Cronbach’s alpha values with a corresponding 95% confidence interval (CI) using the bootstrap method (100 replications). The WHO-5 and PAID scales were not calculated if just one item had missing values. Differences between responders and non-responders of the questionnaire retest (Test 2) were evaluated by X^2^ test or the Kruskal–Wallies test following categorical or continuous variables on available data from the first questionnaire response (Test 1).

Test–retest reliability of single ordinal items was assessed using weighted kappa statistic with squared weights and corresponding 95% CI estimated using the bootstrap method (1000 replications). The interpretation of the kappa coefficients followed the recommendations of Landis et al.: < 0.2 (slight), 0.21–0.4 (fair), 0.41–0.60 (moderate), 0.61–0.8 (substantial), and 0.81–1.0 (almost perfect) [30]. Intraclass correlation coefficients (ICC) absolute-agreement, 2-way mixed-effects model with corresponding 95% CI were used to assess the test–retest reliability of the WHO-5 and PAID scales [31]. An ICC of 0.70 is considered acceptable at the group level; however, at the patient level, an ICC of 0.90 is recommended [8].

Measurement error of the WHO-5 and PAID scales was evaluated using standard error of the measurement. First, the differences between Test 1 and Test 2 were illustrated in a Bland–Altman plot with 95% CI and 95% limits of agreement (LOA). The differences between scores (Test 1 − Test 2) were plotted against the means of the two Test 1 and Test 2 scores ((Test 1 + Test 2)/2)) [8]. LOA equals the mean systematic difference in scores between Test 1 and Test 2 ± 1.96 × standard deviation (SD) of the difference. LOA gives an indication of the size of the measurement error and is expressed in the units of the measurement [8]. Second, the standard error of the measurement (SEM) was estimated, which equals the square root of the error variance, reflecting the intra-individual variation [32]. The interpretation of SEM is not straightforward. Thus, the minimal detectable change (MDC) was calculated based on the SEM. MDC with 95% CI equals 1.96 ± √2 × SEM reflecting the smallest within-person change, which can be explained as a real individual change above the measurement error [32]. Thus, a change in scores smaller than the MDC can be due to measurement error and may not be a real change. All analyses were performed using the Stata software version 17 (StataCorp. 2021. Stata Statistical Software: Release 17. College Station, TX: StataCorp LLC).

## Results

### Participants and item characteristics

During the recruitment period, 255 patients received the annual DiabetesFlex questionnaire. The questionnaire was completed by 231 (91%) patients (Test 1), and 146 (57%) patients completed the retest (Test 2). The median response time between the two-time points was five days, and the interquartile range (IQR) was 5 to 7 days. The mean age was 52.8 SD (13.5) years; 47% were female, and 48% reported excellent/very good general health (Table 1). Non-responders to the second questionnaire were younger than responders, but no differences were found in gender, general health, well-being, or diabetes distress. A total of 145 patients completed the WHO-5 scale at both time points, and 108 patients completed the PAID scale twice. There was a tendency toward ceiling effects in all items in both the WHO-5 and PAID questionnaires (Tables 2, 3), but no missing values were present among completers. Cronbach’s alpha for the WHO-5 was 0.89 (95% CI 0.86–0.93) in Test 1 and 0.90 (95% CI 0.86–0.93) in Test 2. In PAID, Cronbach’s alpha was 0.93 (95% CI 0.92–0.95) in Test 1 and 0.94 (95% CI 0.92–0.96) in Test 2.

**Table 1:** Patients characteristics of responders and non-responders of the second questionnaire (Test 2) based on data from the annual DiabetesFlex questionnaire (Test 1), N = 231

	Respondents	Non-respondents	*P* value
	n = 146	n = 85	
Age, years			
Mean (SD)	52.8 (13.5)	44.9 (13.7)	
Median (IQR)	54.5 (44.4–62.9)	44.4 (34.1–54.6)	*p* = 0.0001
Gender, *n* (%)			
Female	69 (47)	37 (44)	
Male	77 (53)	48 (56)	*p* = 0.58
General health, *n* (%)			
Excellent	5 (3.4)	10 (11.8)	
Very good	65 (44.5)	37 (43.5)	
Good	54 (37.0)	28 (32.9)	
Fair	18 (12.3)	9 (10.6)	
Poor	4 (2.7)	1 (1.2)	*p* = 0.15
Well-being (WHO-5)			
Mean (SD)	66.6 (18.9)	68.5 (17.2)	
Median (IQR)	72 (56–80)	76 (56–80)	*p* = 0.59
Problem Areas in Diabetes (PAID)			
Mean (SD)	14.1 (13.0)	11.6 (11.2)	
Median (IQR)	9.4 (3.8–23.1)	7.5 (3.8–18.8)	*p* = 0.24
Missing, *n*	2	0	

*SD* standard deviation, *IQR* interquartile range, *WHO-5* WHO-Five Well-being Index, *PAID* Problem Areas in Diabetes

**Table 2:** Item level distribution and weighted kappa of the WHO-Five Well-being Index among 145 patients with type 1 diabetes

Item	Item content		Distribution (%) of the response options^a^						Test–retest Weighted kappa
			0	1	2	3	4	5	
1	I have felt cheerful and in good spirits	Test 1	0	7.5	6.9	15.1	61.0	9.6	0.74 (0.58–0.84)
		Test 2	0	4.8	8.9	20.6	58.9	6.9	
2	I have felt calm and relaxed	Test 1	0.7	8.9	8.2	26.0	48.6	7.5	0.75 (0.61–0.85)
		Test 2	0.7	6.2	11.6	21.2	51.4	8.9	
3	I have felt active and vigorous	Test 1	4.1	8.2	13.7	21.9	48.0	4.1	0.79 (0.61–0.87)
		Test 2	3.4	6.9	11.0	32.2	40.4	6.2	
4	I woke up feeling fresh and rested	Test 1	4.8	10.3	13.7	24.0	43.2	4.1	0.80 (0.69–0.87)
		Test 2	7.5	8.2	11.6	24.0	45.2	3.4	
5	My daily life has been filled with things that interest me	Test 1	0.7	6.9	6.2	19.2	56.2	11.0	0.70 (0.54–0.80)
		Test 2	0	7.5	8.2	19.9	57.5	6.9	

^a^0 = at no time, 1 = some of the time, 2 = less than half of the time, 3 = more than half of the time, 4 = most of the time, 5 = all of the time

**Table 3:** Item level distribution and weighted kappa of the Problem Areas in Diabetes (PAID) questionnaire among 108 patients with type 1 diabetes

Item	Item content		Distribution (%) of the response options^a^					Test–retest Weighted kappa
			0	1	2	3	4	
1	Not having clear and concrete goals for your diabetes care	Test 1	68.8	22.0	7.3	1.8	0	0.67 (0.51–0.79)
		Test 2	71.6	23.9	2.8	1.8	0	
2	Feeling discouraged with your diabetes treatment plan	Test 1	67.0	21.1	10.1	0.9	0.9	0.70 (0.52–0.83)
		Test 2	67.9	23.9	6.4	1.8	0	
3	Feeling scared when you think about living with diabetes	Test 1	63.3	29.4	5.5	0.9	0.9	0.76 (0.59–0.86)
		Test 2	61.5	24.8	11.9	1.8	0	
4	Uncomfortable social situations related to your diabetes care (e.g. people telling you what to eat)	Test 1	70.6	22.9	5.5	0.9	0	0.63 (0.33–0.82)
		Test 2	71.6	22.0	5.5	0.9	0	
5	Feelings of deprivation regarding food and meals	Test 1	64.2	29.4	5.5	0.9	0	0.61 (0.45–0.74)
		Test 2	64.2	26.6	9.2	0	0	
6	Feeling depressed when you think about living with diabetes	Test 1	63.3	24.8	10.1	1.8	0	0.74 (0.59–0.86)
		Test 2	67.0	17.4	13.8	0.9	0.9	
7	Not knowing if your mood or feelings are related to your diabetes	Test 1	49.5	26.6	19.3	4.6	0	0.77 (0.66–0.86)
		Test 2	54.1	25.7	16.5	3.7	0	
8	Feeling overwhelmed by your diabetes	Test 1	62.4	23.9	11.9	0.9	0.9	0.80 (0.66–0.89)
		Test 2	62.4	23.9	11.0	0.9	1.8	
9	Worrying about low blood sugar reactions	Test 1	43.1	31.2	20.2	4.6	0.9	0.68 (0.50–0.80)
		Test 2	45.9	34.9	18.4	0	0.9	
10	Feeling angry when you think about living with diabetes	Test 1	73.4	16.5	8.3	1.8	0	0.81 (0.64–0.90)
		Test 2	75.2	16.5	6.4	1.8	0	
11	Feeling constantly concerned about food and eating	Test 1	60.6	30.3	6.4	2.8	0	0.60 (0.39–0.76)
		Test 2	66.1	24.8	8.3	0.9	0	
12	Worrying about the future and the possibility of serious diabetes complications	Test 1	33.0	34.9	19.3	10.1	2.8	0.82 (0.74–0.88)
		Test 2	37.6	24.8	30.3	6.4	0.9	
13	Feelings of guilt or anxiety when you get off track with your diabetes management	Test 1	50.5	29.4	17.4	2.8	0	0.67 (0.52–0.80)
		Test 2	61.5	19.3	16.5	2.8	0	
14	Not ‘accepting’ your diabetes	Test 1	77.1	15.6	5.5	1.8	0	0.70 (0.47–0.84)
		Test 2	78.0	13.8	7.3	0	0.9	
15	Feeling unsatisfied with your diabetes physician	Test 1	83.5	8.3	4.6	3.7	0	0.67 (0.39–0.84)
		Test 2	84.4	10.1	3.7	1.8	0	
16	Feeling that diabetes is taking up too much of your mental and physical energy every day	Test 1	42.2	31.2	19.3	5.5	1.8	0.71 (0.57–0.81)
		Test 2	45.0	34.9	15.6	4.6	0	
17	Feeling alone with your diabetes	Test 1	63.3	23.9	11.9	0.9	0	0.72 (0.57–0.83)
		Test 2	67.9	18.4	13.8	0	0	
18	Feeling that your friends and family are not supportive of your diabetes management efforts	Test 1	76.2	15.6	7.3	0	0	0.71 (0.48–0.87)
		Test 2	83.5	11.9	4.6	0	0	
19	Coping with complications of diabetes	Test 1	56.9	25.7	14.7	1.8	0.9	0.73 (0.57–0.84)
		Test 2	57.8	22.9	17.4	0.9	0.9	
20	Feeling ‘burned out’ by the constant effort needed to manage diabetes	Test 1	51.4	27.5	11.9	7.3	1.8	0.66 (0.52–0.78)
		Test 2	50.5	33.9	9.2	6.4	0	

^a^0 = not a problem, 1 = minor problem, 2 = moderate problem, 3 = somewhat serious problem, 4 = serious problem

### Missing data and reasons for non-response to the second questionnaire (Test 2)

Among the responders to the second questionnaire, one patient (0.7%) and 38 patients (26%) did not answer the WHO-5 or PAID questionnaires, respectively. Furthermore, missing data in single items ranged from 0.7% in the general health status item to 25.3% in the decreased feet feeling and pain in feet items. The highest proportion of missing data was found in the erectile dysfunction item (60.3%) (Table 5). Informal conversations with patients related to non-response indicated two primary reasons for not completing the second questionnaire: 1). Some patients did not understand the purpose of the second questionnaire as they had already answered the same questionnaire at test 1, and 2). Some patients expressed a lack of energy to fill in an additional questionnaire.

### Test–retest reliability and measurement error of the WHO-Five Well-being Index

**Fig. 1:**
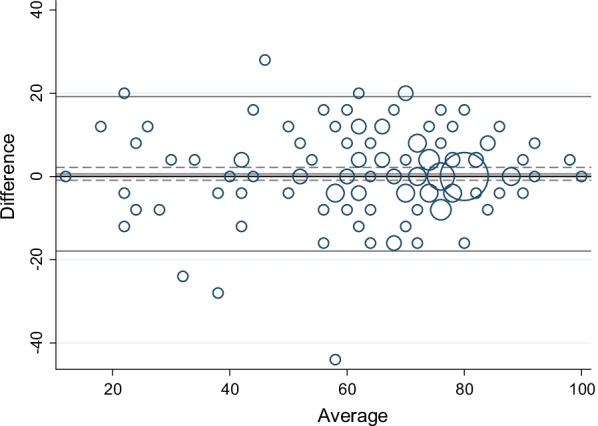
Differences in the WHO-Five Well-being Index scale between first and second questionnaire responses (Test 1 and Test 2) plotted against the mean, N = 145

Weighted kappa values for the five ordinal items included in the WHO-5 scale were overall substantial (Table 2). As shown in Table 4, the ICC of the WHO-5 scale was 0.87 (95% CI 0.82–0.90). Figure 1 presents the differences between Test 1 and Test 2 plotted against the mean of the two measurement time points. The standard error of the measurement was 6.70 (95% CI 6.01–7.57), and the calculated MDC was 18.56 points (95% CI 16.65–20.99).

**Table 4:** Test–retest reliability and measurement error of the WHO-Five Well-being Index (WHO-5) and the Problem Areas in Diabetes (PAID) scale between Test 1 and Test 2

	WHO-5	PAID
N	145	108
Mean (95% CI) Test 1	66.98 (63.94–70.02)	14.36 (11.87–16.86)
Mean (95% CI) Test 2	66.34 (63.34–69.35)	13.07 (10.57–15.57)
Difference (95% CI)	0.63 (− 0.92–2.19)	1.30 (0.14–2.45)
SEM (95% CI)	6.70 (6.01–7.57)	4.28 (3.78–4.94)
MDC (95% CI)	18.56 (16.65–20.99)	11.86 (10.46–13.70)
ICC (95% CI)	0.87 (0.82–0.90)	0.89 (0.84–0.92)

*CI* confidence interval, *SEM* standard error of the measurement, *MDC* minimal detectable change, *ICC* intra class correlation coefficient

### Test–retest reliability and measurement error of the PAID questionnaire

**Fig. 2:**
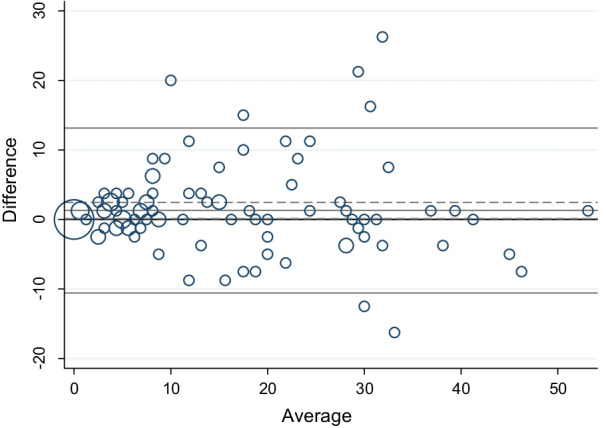
Differences in the Problem Areas in Diabetes (PAID) scale between first and second questionnaire responses (Test 1 and Test 2) plotted against the mean, N = 108

Weighted kappa values for the 20 ordinal items included in the PAID scale were moderate to substantial (Table 3). As shown in Table 4, the ICC of the PAID scale was 0.89 (95% CI 0.84–0.92). Figure 2 presents the differences between Test 1 and Test 2 plotted against the mean of the two measurement time points. The standard error of the measurement was 4.28 (95% CI 3.78–4.94), and the calculated MDC was 11.86 points (95% CI 10.46–13.70).

### Test–retest reliability of single symptom and general health items

Table 5 shows the test–retest reliability estimates of the 11 ordinal single symptom and general health items included in the DiabetesFlex questionnaire. The estimates were substantial for eight items, with weighted kappa values ranging from 0.79 (95% CI 0.70–0.87) (rapid heartbeat) to 0.90 (95% CI 0.78–0.98) (erection problems). Broad confidence intervals were observed in the symptom items regarding pain in the leg when walking, foot ulcer, and decreased foot feeling; thus, these items showed fair to substantial test–retest reliability, weighted kappa values were 0.77 (95% CI 0.56–0.91), 0.72 (95% CI 0.22–0.94), and 0.73 (95% CI 0.44–0.91), respectively.

**Table 5:** Test–retest reliability of single symptom and general health items among 146 patients with type 1 diabetes

Item	n	Levels	Agreement/expected agreement %	Weighed kappa (95% CI)
General health	145	5	98.3/91.2	0.81 (0.73–0.88)
General health last year	146	5	98.7/93.8	0.79 (0.71–0.88)
Hypoglycemia	113	5	98.2/88.5	0.84 (0.72–0.91)
Rapid heartbeat	112	5	98.1/91.1	0.79 (0.70–0.87)
Dyspnea	112	5	98.8/92.3	0.84 (0.70–0.92)
Heart chest pain	112	5	99.1/93.1	0.87 (0.73–0.95)
Pain leg walking	112	5	97.7/89.9	0.77 (0.56–0.91)
Foot ulcer	112	5	98.9/96.0	0.72 (0.22–0.94)
Decreased feeling feet	109	5	98.3/93.9	0.73 (0.44–0.91)
Pain feet	109	5	98.9/90.8	0.88 (0.80–0.93)
Erection problems	58	5	97.6/75.4	0.90 (0.78–0.98)

*CI* confidence interval

## Discussion

The WHO-Five Well-being Index and the Problem Areas in Diabetes (PAID) questionnaire used in a PRO-based telehealth intervention in clinical practice showed substantial test–retest reliability among patients with type 1 diabetes. Many of the single items measuring symptoms and general health also showed substantial test–retest reliability. The measurement error of the WHO-5 and PAID questionnaires varied, and a larger measurement error was found in the WHO-5 scale than in the PAID scale. The estimated MDC was 18.60 points in WHO-5 and 11.90 points in PAID.

Few other studies have investigated the reliability in terms of test–retest reliability and measurement error of the WHO-5 and PAID questionnaires. However, reliability in terms of internal consistency of both questionnaires has been documented by several studies across patient populations and countries. We found a Cronbach’s alpha of 0.89 on the WHO-5 scale, which corresponds to other studies’ findings [33–36]. Furthermore, we found a Cronbach’s alpha of 0.93 for the PAID questionnaire, which parallels other studies’ findings [21, 22, 37].

Supporting our finding, a Danish study among outpatients with epilepsy also found substantial test–retest reliability of the WHO-5 scale [13]. The study identified an MDC of 22.31 points of the WHO-5 scale in web responders, supporting our finding of an MDC of 18.56 points [13]. The size of the MDC is considered imperative if the WHO-5 scale is used to measure change over time at the individual level in clinical practice since a WHO-5 change score lower than 18.56 points may be due to measurement error and not a real change. A Spanish study evaluated the test–retest reliability of the WHO-5 scale in patients with bipolar disorder [12]. They reported a reliability correlation coefficient of 0.83 of the WHO-5 scale; however, the measurement error was not reported [12]. Furthermore, a German study also assessed the test–retest reliability of the WHO-5 scale among patients with rheumatoid arthritis [11]. They found a lower reliability correlational estimate than expected a priori, and they did not report the measurement error [11]. The findings from the Spanish and German studies are not directly comparable to our study, as we used ICC to measure the test–retest reliability estimates in a different patient population.

A Norwegian study of patients with type 1 and type 2 diabetes found high test–retest reliability of the PAID questionnaire. They found an ICC of 0.79 compared to 0.89 in our study [19]. Furthermore, a study from Korea found an ICC of 0.89 (0.83–0.94) [37], and a study from China reported a reliability estimate of 0.83 [26]. These results parallel our findings; however, the studies were conducted only among patients with type 2 diabetes. Measurement error was not reported by other studies that evaluated the PAID questionnaire’s reliability, which underlines the importance of this finding in our study. The lack of measures of reliability and measurement error in the PAID questionnaire is pointed out as an issue by a recently published systematic review regarding diabetes distress instruments, which supports the need for further research [22].

We used 11 single items that aimed to measure clinically relevant symptoms and general health. The two items measuring general health were selected from the SF-36 [27, 38]. We found reliability estimates of 0.81 and 0.79 in the global general health status and the 1-year retrospective general health status items, respectively. A study has found a lower reliability estimate of 0.51 in the SF-36 general health status item in an US general population [39], and no studies assessing the reliability of the retrospectively general health item have been identified. The other single items in this study were developed by clinical experts in endocrinology and experts in PRO. Content and face validity were ensured during the development process; however, measurement properties were not further evaluated until this study. Reliability is only one relevant measurement property, and we are aware of the need to evaluate other properties, such as validity and responsiveness, in future research.

This study followed the COSMIN’s recommendation about evaluating measurement properties such as reliability and measurement error of a PRO instrument [28, 29]. However, some of the strengths and limitations of this study need to be further elaborated. The enrollment of patients in our study is considered adequate, but the response rate at the second measurement time point was only 57%. Potential selection bias exists, but as shown in Table 1, responders did not differ regarding general health and mental well-being compared to non-responders, supporting a heterogeneous study population. However, we cannot exclude differences between responders and non-responders in unmeasured disease-related aspects, such as long-term complications and co-morbidity. This study's age and gender distribution was close to what has been reported in other studies among patients with type 1 diabetes in Denmark [7, 40]. However, the WHO-5 score has been reported to be lower [40] and higher [7] in other studies.

The COSMIN checklist highlights three important design requirements in a test–retest study. First, to ensure stability in patients’ health conditions between the two measurement time points. Second, to select an appropriate time interval between the two measurements. Third, to ensure similar test conditions at the two measurement time points [29, 41]. We selected a relatively short time interval because the study participants had scheduled in-clinic appointments. We aimed to ensure that the patients filled in the second questionnaire before the in-clinic visit at the hospital. The median interval between the two measurement time points was only five days. Choosing a short time interval could have introduced recall bias if the patients remembered their answers at the first time point; however, the risk of a change in the patients’ health status was reduced. We did not measure whether the participants experienced a real change in their health status between the two measurement time points. This could be done based on measurements of similar well-known reliable constructs concurrently or by including a question regarding a change in health status in the second questionnaire. However, the risk of a real change in the patients’ health status was considered low in our study due to the short time interval between the two measurements. Finally, similar test conditions were ensured, since all patients filled in an electronic version of the questionnaires on the same platform. This study only represented patients who were able to respond electronically. To ensure a higher degree of participation equality, it is often recommended to offer different modes of administration or to let family or caregivers have the ability to report on behalf of the patients [42, 43]. This topic is an important future perspective if considering expanding the DiabetesFlex target population to patients with type 2 diabetes.

## Conclusion

The Danish version of the WHO-Five Well-being Index (WHO-5) and the Problem Areas in Diabetes (PAID) questionnaire used in identifying mental health status and diabetes distress among an outpatient type 1 diabetes population showed substantial test–retest reliability. Measurement error of the PAID questionnaire was considered acceptable; however, a larger measurement error of the WHO-5 questionnaire was observed. Further research assessing the reliability and measurement error of both instruments in patients with diabetes and other chronic conditions is considered imperative. In addition, substantial test–retest reliability was found in the single items measuring clinically relevant symptoms and general health; however, the symptom items need to be further validated.

## Data Availability

An anonymous version of the datasets used and analyzed in this study are available from the corresponding author on reasonable request.

## Abbreviations

PRO: Patient-reported outcome
WHO-5: WHO-Five Well-being Index
PAID: Problem Areas in Diabetes
SF-36: The Short Form 36 Health Survey
COSMIN: Consensus based standards for the Selection of health Measurement Instruments
ICC: Intraclass correlation coefficients
LOA: Limits of agreement
SEM: Standard error of the measurement
MDC: Minimal detectable change
CI: Confidence interval
SD: Standard deviation
IQR: Interquartile range
